# MicroRNA–mRNA Networks in Pregnancy Complications: A Comprehensive Downstream Analysis of Potential Biomarkers

**DOI:** 10.3390/ijms22052313

**Published:** 2021-02-25

**Authors:** Asghar Ali, Frieder Hadlich, Muhammad W. Abbas, Muhammad A. Iqbal, Dawit Tesfaye, Gerrit J. Bouma, Quinton A. Winger, Siriluck Ponsuksili

**Affiliations:** 1 Leibniz Institute for Farm Animal Biology, Institute of Genome Biology, 18196 Dummerstorf, Germany; Hadlich@fbn-dummerstorf.de (F.H.); iqbal@fbn-dummerstorf.de (M.A.I.); ponsuksili@fbn-dummerstorf.de (S.P.); 2 Animal Reproduction and Biomedical Laboratory, Department of Biomedical Sciences, Colorado State University, Fort Collins, CO 80523, USA; Dawit.Tesfaye@colostate.edu (D.T.); Gerrit.Bouma@colostate.edu (G.J.B.); quinton.winger@colostate.edu (Q.A.W.); 3 Department of Bioinformatics and Biotechnology, Government College University, Faisalabad 38000, Pakistan; abbas.waseem.gcu@gmail.com

**Keywords:** preeclampsia, intrauterine growth restriction, biomarkers, miRNAs, miR-210

## Abstract

Pregnancy complications are a major cause of fetal and maternal morbidity and mortality in humans. The majority of pregnancy complications initiate due to abnormal placental development and function. During the last decade, the role of microRNAs (miRNAs) in regulating placental and fetal development has become evident. Dysregulation of miRNAs in the placenta not only affects placental development and function, but these miRNAs can also be exported to both maternal and fetal compartments and affect maternal physiology and fetal growth and development. Due to their differential expression in the placenta and maternal circulation during pregnancy complications, miRNAs can be used as diagnostic biomarkers. However, the differential expression of a miRNA in the placenta may not always be reflected in maternal circulation, which makes it difficult to find a reliable biomarker for placental dysfunction. In this review, we provide an overview of differentially expressed miRNAs in the placenta and/or maternal circulation during preeclampsia (PE) and intrauterine growth restriction (IUGR), which can potentially serve as biomarkers for prediction or diagnosis of pregnancy complications. Using different bioinformatics tools, we also identified potential target genes of miRNAs associated with PE and IUGR, and the role of miRNA-mRNA networks in the regulation of important signaling pathways and biological processes.

## 1. Introduction:

### 1.1. Placenta and Pregnancy Complications

Successful placentation is the critical step for the successful establishment and optimal outcome of pregnancy. Once established, the placenta provides nutrients and oxygen to the growing fetus, removes waste products, metabolizes different nutrients and releases metabolic products in both fetal and maternal circulation, and produces and releases hormones in both fetal and maternal circulation, which are important for metabolism and fetal growth [1]. In addition to being a multifunctional organ, the placenta is also highly capable of structural and functional adaptations and mitigates the effects of insults of maternal origin and maintains a conducive intrauterine environment for the developing fetus [2]. Human placental development starts when the trophectoderm of hatched blastocyst interacts with the uterine epithelium at around 6–7 days post-fertilization [3]. The key events of placental development include differentiation of cytotrophoblasts cells into extravillous trophoblast cells (EVTs) and syncytiotrophoblast, invasion and migration of EVTs, and remodeling of maternal spiral arteries [3,4]. Humans have a hemochorial placenta, where syncytiotrophoblast is in direct contact with maternal blood and is responsible for the exchange of gases, nutrients, metabolites, and wastes between fetal and maternal circulations [5,6,7]. During the early stages of placental development, the dynamic turnover of trophoblasts is tightly regulated by complex molecular pathways to ensure normal placental development and function [4]. An imbalance between trophoblast proliferation and differentiation can result in severe placental pathologies and pregnancy complications [8]. Therefore, it is important to uncover the molecular mechanisms involved in trophoblast turnover and function.

Impairment of placental function can result in serious pregnancy complications including intrauterine growth restriction, preeclampsia, preterm birth, gestational diabetes, gestational hypertension, and early abortion [9]. Pregnancy complications affect prenatal fetal and maternal health and can have long-lasting postnatal effects due to impaired fetal developmental programming [2]. Intrauterine growth restriction (IUGR) and preeclampsia (PE) are the most prevalent pregnancy complications [10]. Every year, approximately 10–15% of pregnancies are affected by IUGR [11]. IUGR is characterized by the failure of the fetus to reach its genetically determined growth potential and fetal weight less than the 10th percentile for gestation age [12]. The terms IUGR and fetal growth restriction (FGR) are used interchangeably and both refer to a diagnosis of growth restriction before delivery; however, there is still no clear consensus on the definition of these terms [13]. Newborns with IUGR are at a higher risk of developing serious health disorders, including impaired neurological development and metabolic syndrome in adulthood [12,14]. Although the majority of IUGR cases are idiopathic, several IUGR cases are caused due to impaired placental development and function, especially impairment of placental angiogenesis and insufficient nutrient supply to the fetus [15,16,17]. Placental ability to ensure sufficient blood and nutrient supply to the fetus depends on placental size, placental morphology, placental angiogenesis, uteroplacental and fetoplacental blood flow, and expression level of different nutrient transporters in the placenta [18]. Moreover, placental size and weight are significantly reduced in IUGR pregnancies compared to age-matched healthy pregnancies [19]. Preeclampsia (PE) is a leading cause of maternal and fetal morbidity and mortality and affects 5–8% of pregnancies worldwide [20]. It is clinically diagnosed after week 20 of gestation and is characterized by maternal hypertension and proteinuria. PE poses high risks to both mother and fetus. In mothers, it can affect multiple systems and increases the risk of diabetes mellitus, hypertension, cardiovascular, liver and brain problems [20,21]. The potential effects of PE on fetuses include preterm birth, growth restriction, prenatal death, and long-term cardiovascular disorders [20,21,22]. Similar to many other pregnancy complications, the root cause of PE is also the placenta and its symptoms begin to abate with the delivery of the placenta [20,21]. The main placental pathologies leading to preeclampsia are impaired spiral artery remodeling and reduced placental perfusion [23]. Another important factor to keep in consideration is the difficulty in timely and appropriate diagnosis of IUGR and particularly PE, which is mostly diagnosed during the third trimester of pregnancy [24,25]. An early prediction of PE and IUGR can greatly help to improve the management of these disorders. Therefore, it is of utmost importance to better understand molecular mechanisms involved in placental development and find reliable potential early biomarkers of pregnancy complications.

### 1.2. MicroRNAs as Biomarkers of Pregnancy Complications

MicroRNAs (miRNAs) are small non-coding RNAs that are 22 nucleotides long and have emerged as major regulators of prominent genetic pathways involved in different biological and pathological processes [4,26]. MiRNAs find their complementary target sites in the target mRNA and cause their degradation or translational inhibition [27]. MiRNAs bind in the 3′-untranslated region (3’-UTR) of more than 50% protein-coding mRNAs in humans [28], and there is growing evidence that miRNAs can also repress gene expression by binding in 5´-UTR and coding regions of target mRNAs [29]. Therefore, it can be safely stated that miRNAs are the most prevalent posttranscriptional regulators of gene expression.

The human placenta expresses several miRNAs, and their expression is regulated by different factors including hypoxia, environmental factors, and epigenetic modifications [26,30,31]. MiRNAs play critical roles in placental development and function by regulating the expression of genes involved in trophoblast cell proliferation, differentiation, invasion, migration, apoptosis, and angiogenesis [4]. Aberrant miRNA expression can interfere with trophoblast function and angiogenesis, and result in severe pregnancy complications [4]. Several miRNAs in the placenta have been associated with different pregnancy complications including IUGR, early pregnancy loss, PE, gestational diabetes mellitus, and preterm birth [30,32]. Because the placenta releases DNA and RNA molecules in maternal circulation, the miRNAs of fetal or placental origin can be detected in maternal circulation and can also affect maternal health [33,34]. Expression of miRNAs in the placenta is also affected by the nutritional status of mothers, maternal obesity, pre-pregnancy body mass index (BMI), and gestational weight gain in mothers [35,36,37]. Different studies have shown that placental miRNAs are produced in trophoblast cells, and can be exported to both fetal and maternal circulation [38,39,40]. Therefore, differential expression of a miRNA in maternal circulation can be indicative of underlying placental pathology and may serve as a biomarker for early detection of pregnancy complications. In this study, we reviewed the miRNAs that are differentially expressed in the placenta or maternal circulation during pregnancy and analyzed their target genes and their potential involvement in maternal and fetal physiology.

## 2. Identification of miRNA–mRNA Networks Linked to Placental Development

### 2.1. MicroRNA Inclusion Criteria

Different studies have linked differential expression of miRNAs with reproductive disorders. All studies included in this review presented data from human subjects who had developed symptoms of PE or IUGR and used samples from age-matched healthy pregnant women at the same stage/trimester of pregnancy as control. All studies showing differential expression of miRNAs in preeclamptic or IUGR placentas used the term placental tissues collected at the time of delivery. With exception of few miRNAs, all circulating miRNAs included in this study were measured in blood samples collected during the third trimester at the time of diagnosis or delivery. We shortlisted the miRNAs whose differential expression in placenta and/or maternal circulation was validated through quantitative real-time PCR (qRT-PCR), except some PE-linked circulating miRNAs reported by Yang et al. (2011) [41]. The gestational age (GA) at the time of sampling and its comparison between healthy and complicated pregnancies, maternal age for both healthy and complicated pregnancies, and type of sample (placenta, serum, or plasma) for the studies included in this review are listed in Table S5. Using the data provided in individual studies, we compared gestational ages both at the time of sampling and at the time of delivery between different groups; healthy versus PE and healthy versus IUGR pregnancies (Table S5). For all studies conducted during the first or second trimester, the blood samples were collected at the same GA. In the studies conducted during the third trimester, GA in preeclamptic patients was significantly low (*p* < 0.05) compared to healthy controls both at the time of sampling and at the time of delivery. However, there was no significant difference in GA at the time of sampling (*p* = 0.18) or delivery (*p* = 0.14) in IUGR pregnancies compared to healthy controls. In the majority of cases, PE is accompanied by preterm birth [42], which explains the significantly reduced gestational age in preeclamptic pregnancies included in our study. All PE- and IUGR-linked miRNAs meeting our inclusion criteria are listed in Table 1 and Table 2, respectively. All PE- or IUGR-linked miRNAs that we used for downstream analysis were reported to be upregulated in the placenta or maternal circulation at around the same gestational age in different studies (at the time of delivery or third trimester). To find the target genes of miRNAs, we used two sources to get the lists of genes involved in placental development and functions. Azar et al. isolated human placental cytotrophoblasts from placentas of uncomplicated term pregnancies [43]. Cytotrophoblasts were plated and by 48 h, multinucleated syncytiotrophoblast phenotype was prominent (>85%) [43]. They harvested cytotrophoblasts (24 h) and syncytiotrophoblast (72 h) for RNA extraction and analysis. They found that 5482 genes were differentially expressed (False Discovery Rate (FDR) < 0.05), indicating potential involvement of these genes in trophoblast function and turnover [43]. It was used as List 1 of genes used in this study (Table S1). Because these genes are differentially expressed during syncytial differentiation of cytotrophoblasts, the information from their downstream analysis may not be related to the differentiation of cytotrophoblasts to EVTs. According to the human protein atlas, 494 genes are elevated in the human placenta compared to all other tissues in the body, and it was used as List 2 of genes in this study (Table S2) [44]. The potential targets of shortlisted miRNAs were identified in the genes Lists 1 and 2. 

### 2.2. Identification of miRNA Target Genes

Sequences for each gene’s 3´-UTR, 5´-UTR, and coding regions (coding sequences [CDSs]) were generated from Ensembl annotation 102 (Cambridge, UK) based on reference genome *Homo_sapiens.GRCh38*, and sliced to a maximum of 2000 base pairs sequence length with 50 bases overlap. These sequences were tested for being potential microRNA targets using RNA hybrid version 2.1.2 with parameters for single hit per target, human-based assumed *p*-value distribution, minimum free energy (MFE) threshold of <–25 kcal/mole, and helix constraint from base 2 to 7 [121,122].

### 2.3. Downstream Functional Analysis

We selected miRNA–mRNA pairs with minimum free energy < –25 kcal/mole and *p*-value < 0.05 for further downstream functional analysis. All miRNA–mRNA pairs are listed in Table S3 and S4. We identified target genes of 17 potential early biomarkers of PE (miR-21, miR-122, miR-155, miR-181a, miR-125b, miR-210, miR-193b, miR-516b, miR-518b, miR-221, miR-520a, miR-141, miR-136, miR-29a, miR-423, miR-152, and let-7c) from genes in List 2. These target genes of 17 miRNAs were used for downstream gene ontology analysis for biological processes and Kyoto Encyclopedia of Genes and Genomes (KEGG) pathways enrichment analysis using ClueGO (version 2.5.1) and Cluepedia (version 1.5.7) plugin in Cytoscape (version 3.8.2) environment [123,124,125]. Similarly, for IUGR, three miRNAs were upregulated in both placenta and maternal circulation. Additionally, miR-210 and miR-193b were upregulated only in the placenta and had multiple targets compared to other miRNAs. Therefore, the genes from List 2 that were identified as potential targets of these five IUGR-linked miRNAs were used for downstream gene ontology analysis for biological processes and KEGG pathways enrichment analysis. The parameters used for ClueGO analysis were a hypergeometric test that was used for enrichment analysis and Benjamini–Hochberg correction was used for multiple testing correction and the *Homo sapiens* genome assembly as a reference. ClueGO generates functionally annotated KEGG pathways and gene ontologies of miRNAs and their downstream targets. Pie charts for KEGG pathways and biological processes of miRNA-target genes from List 1 were made using Protein Analysis Through Evolutionary Relationships (PANTHER) classification system (v.16.0) [126]. The KEGG pathways and gene ontologies that passed the *p*-value threshold (*p* < 0.05) were considered significantly enriched.

## 3. Findings and Discussion

### 3.1. MicroRNAs in PE and IUGR

**Figure 1:**
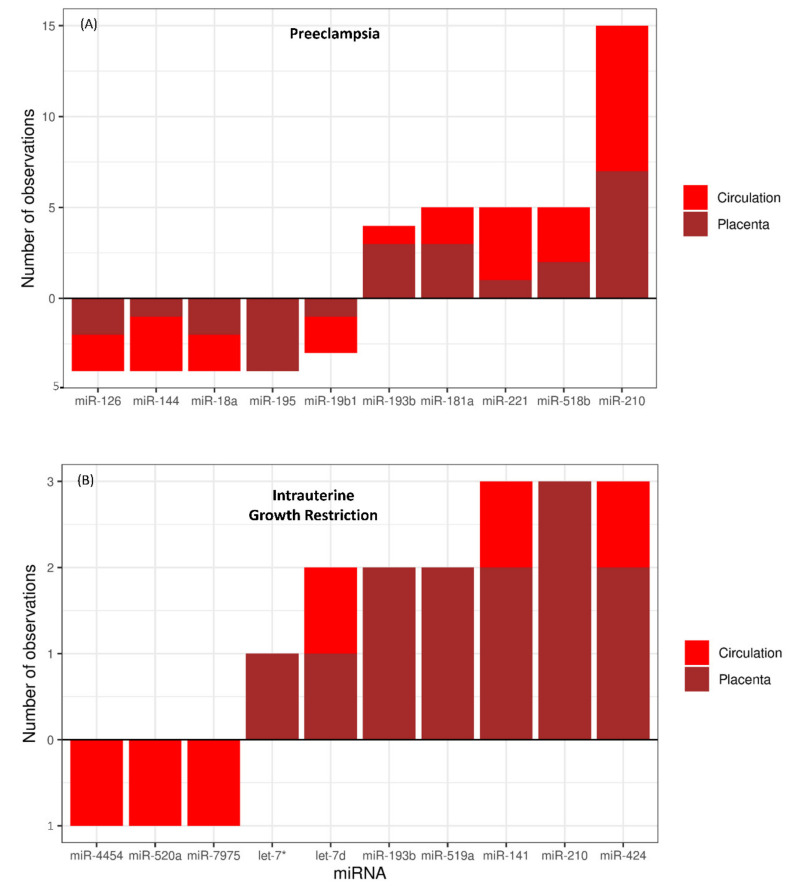
(**A**) Number of observations linking upregulated and downregulated miRNAs to PE. A total of 15 different studies linked upregulation of miR-210 with the pathogenesis of PE, eight times in circulation and seven times in the placenta. (**B**) Number of observations linking upregulated and downregulated miRNAs to IUGR. Three different studies linked upregulation of miR-424 with the pathogenesis of IUGR, one time in circulation and two times in the placenta. Bars rising above zero *x*-axis reference line indicate upregulation, and bars hanging below zero *x*-axis reference line indicate downregulation. A complete list of miRNAs and the number of studies linking them to each disease is in Table S5. Let-7*: let-7a, let-7b, let-7c, let-7e, let-7f, let-7g or let-7i.

Several studies have linked miRNAs with the pathophysiology of IUGR and PE. In the studies reviewed in this paper, 56 studies reported 127 different miRNAs that were differentially expressed in the placenta and/or maternal circulation in pregnancies complicated with PE (Table 1; Table S5). Upregulation of miR-210 was reported in 15 studies (eight in maternal circulation and seven in placental tissue). Other miRNAs that were upregulated in PE in five independent studies for each miRNA include miR-181a, miR-221, and miR-518b (Figure 1A). Similarly, downregulation of miR-126, miR-144, miR-18a, and miR-195 in the placenta and/or maternal circulation was reported in 4 different studies for each of these miRNAs (Figure 1A). Out of 127 PE-linked miRNA, 37 miRNAs were upregulated only in maternal circulation, 27 were upregulated only in the placenta, and 15 (miR-21, miR-122, miR-155, miR-181a, miR-125b, miR-210, miR-193b, miR-516b, miR-518b, miR-221, miR-520a, miR-141, miR-136, miR-29a, and miR-152) were upregulated in both placenta and maternal circulation. Similarly, 8 miRNAs were downregulated only in maternal circulation, 27 miRNAs were downregulated only in the placenta, and 6 miRNAs (miR-18a, miR-223, miR-19b1, miR-144, miR-126, and miR-92a1) were downregulated in both placenta and maternal circulation. We also found some inconsistencies in the regulation of few miRNAs linked to PE. For example, miR-542, miR-423, and let-7c were reported to be downregulated in the placenta but upregulated in maternal circulation in different studies [41,62,88]. Similarly, let-7d was reported to be downregulated in circulation but upregulated in the placenta [41,72], whereas miR-584 and miR-17 have been found to be both downregulated and upregulated in the placenta in different studies [66,73,85,88]. These inconsistencies in miRNA dysregulation in different studies are hard to explain. Different preeclamptic patients can have different predisposing factors and different types or severity of placental pathology, which might be the possible reasons for inconsistent regulation of certain miRNAs during the pathogenesis of PE. The frequency at which each miRNA was found to be differentially expressed in the placenta or maternal circulation during PE is listed in Table S5.

We found 24 independent studies that reported differential expression of 76 different miRNAs in maternal circulation and/or placenta in pregnancies complicated with IUGR (Table 2; Table S5). Only six miRNAs including let-7d, miR-193b, miR-519a, miR-141, miR-210, and miR-424 have been linked to IUGR in more than one independent study (Figure 1B). Upregulation of three miRNAs (miR-141, miR-424, and let-7d) has been reported both in maternal circulation and placenta from pregnancies complicated with IUGR (Figure 1B). Moreover, downregulation of only 3 miRNAs (miR-4454, miR-520a, and miR-7975) in maternal circulation was linked to IUGR (Figure 1B). We also observed a contradiction in miR-518b regulation, which was reported to be downregulated in maternal circulation but upregulated in the placenta in pregnancies complicated by IUGR [102,120]. The frequency at which each miRNA was found to be differentially expressed in the placenta or maternal circulation during IUGR is listed in Table S5.

**Figure 2:**
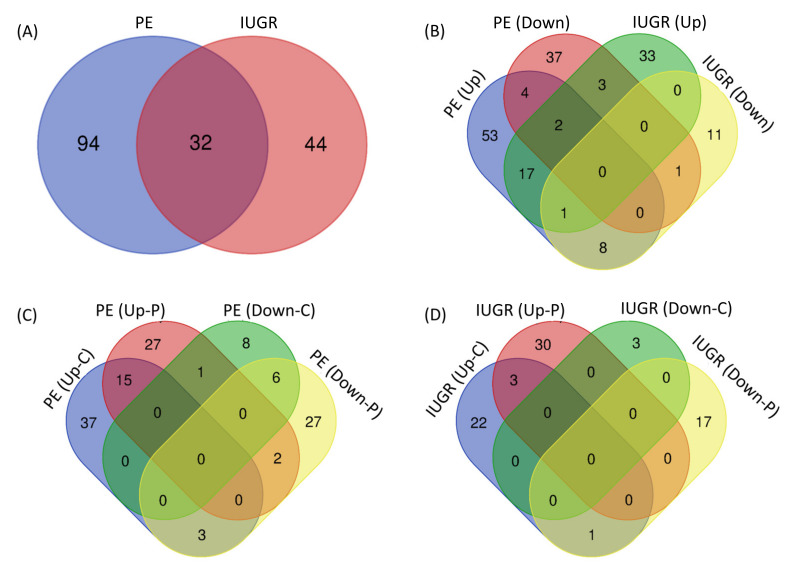
Comparison of total miRNAs linked to PE and IUGR (**A**), upregulated and downregulated miRNAs in PE and IUGR (**B**), different groups of miRNAs linked to PE (**C**), and different groups of miRNAs linked to IUGR (**D**). PE, preeclampsia; IUGR, intrauterine growth restriction; C, circulation; P, placenta.

Comparison of all differentially expressed miRNAs between PE and IUGR shows that differential expression of 94 miRNAs is linked to only PE, differential expression of 44 miRNAs is linked to only IUGR, and differential expression of 32 miRNAs is linked to both IUGR and PE (Figure 2A). Comparison of the expression pattern of these miRNAs shows that 18 miRNAs are upregulated and one miRNA (miR-1) is downregulated in both PE and IUGR (Figure 2B). Out of 127 PE-linked miRNAs, 38 were upregulated only in maternal circulation, 26 were upregulated only in the placenta, and 15 were upregulated in both placenta and maternal circulation (Figure 2C). Out of 76 miRNAs linked to the pathophysiology of IUGR, 22 were upregulated only in maternal circulation, 30 were upregulated only in the placenta, and three were upregulated in both placenta and circulation (Figure 2D). All comparisons are further explained in Figure 2 and Table S5. Most interestingly, miR-210, which is most often linked to the pathogenesis of PE, was also reported to be upregulated in placentas from IUGR pregnancies in three independent studies. Therefore, we will put more emphasis on the role of miR-210 in the regulation of different biological processes and genetic pathways.

Identification of several different miRNAs and their differential expression in the placenta highlights their association with placental health and diseases and shows the complexity of signaling pathways involved in placental development. Some miRNAs that are differentially expressed in the placenta during PE or IUGR, have also been reported to be differentially expressed in maternal circulation. For instance, 15 upregulated and six downregulated PE-linked miRNAs in the placenta were also differentially expressed in maternal circulation (Table S5). Similarly, in IUGR, three upregulated miRNAs in the placenta were also differentially expressed in maternal circulation (Table S5). Differential expression of placental miRNAs in maternal circulation during pregnancy complications also indicates the involvement of these miRNAs in maternal morbidity during these disorders [26]. Existing evidence of export of placental miRNAs to feto-maternal compartments [127,128] suggests that dysregulation of miRNAs during placental pathologies can also affect maternal physiology and fetal growth and development. However, the physiological significance of most placenta-originated miRNAs in fetal and maternal compartments remains to be established. The regulation of gene expression by miRNAs is not just a transient phenomenon, but the miRNAs can affect epigenetics machinery in the cells and affect gene expression for a longer period of time [129]. Hence, altered expression of miRNAs in the fetus can change its developmental programing and pose long-lasting effects even after birth [128]. This phenomenon might also be playing an important role in fetal origins of adult diseases, commonly known as “Barker’s hypothesis” [130].

### 3.2. MicroRNA–mRNA Networks in PE

miRNAs regulate gene expression by binding their target mRNAs to cause post-transcriptional or translational repression [27]. Although it is 22 nucleotides long, successful target recognition by a miRNA requires the matching of only eight nucleotide seed sequences with the target mRNA [131]. A single miRNA can target hundreds of genes and accordingly one gene can be targeted by several different miRNAs [132,133]. As a result, a single miRNA can regulate several genetic pathways and biological processes [132,133]. Therefore, differential expression of only one miRNA at a critical stage of placental or fetal development can result in severe pathologies. Using RNA hybrid, we found potential targets of all miRNAs linked to preeclampsia using genes provided in Table S1 (List 1; differentially expressed genes between cytotrophoblasts and syncytiotrophoblast) and Table S2 (List 2; genes with high expression in the placenta). We were surprised to find that miR-638, which is upregulated in the PE placenta, can potentially target 2898 genes from List 1 and 293 genes from List 2. It reasserts that dysregulation of a single miRNA can target multiple genes and affect multiple pathways. Similarly, miR-210, which is linked to both PE and IUGR, can potentially target 968 genes from List 1 and 90 genes from List 2. Targets of all miRNAs associated with PE are provided in Table S3 and S4.

The most critical factor in the pathophysiology of PE is abnormal angiogenesis. Some important genes with well-established roles in placental angiogenesis include vascular endothelial growth factor-A (VEGF-A), VEGF-B, Fms-related tyrosine kinase 1 (FLT1), and mitogen-activated protein kinase (MAPK) [134,135,136]. It is important to mention that these four genes are not the only genes involved in placental angiogenesis. According to our results, these genes are targeted by multiple miRNAs linked to the pathogenesis of PE. Out of 127 PE-linked miRNAs, VGEF-A can be targeted by 10 different miRNAs, VEGF-B can be targeted by 9 different miRNAs, FLT1 is a potential target of 16 different miRNAs, and different isoforms of MAPK can be targeted by 28 different miRNAs (Table S6). These four genes (VEGF-A, VEGF-B, FLT1, and MAPK) are common targets of six different miRNAs, including miR-1247, miR-27a-star, miR-328, miR-638, miR-766, and miR-1233. In conclusion, several miRNAs are associated with the pathophysiology of PE by regulating genetic pathways related to angiogenesis.

Regarding the miRNAs that are differentially expressed only in circulation, their direct involvement in placental pathologies might not be consistent. Similarly, miRNAs differentially expressed only in the placenta, but not in maternal circulation, cannot be used as a non-invasive biomarker. The most interesting miRNAs, with greater potential to be used as PE biomarkers, are the 15 miRNAs (miR-21, miR-122, miR-155, miR-181a, miR-125b, miR-210, miR-193b, miR-516b, miR-518b, miR-221, miR-520a, miR-141, miR-136, miR-29a, and miR-152) that have been reported to be upregulated both in the placenta and maternal circulation in pregnancies complicated by PE. Additionally, miR-423 and let-7c are also potential early PE biomarkers because they have been reported to be upregulated during first trimesters and third trimesters of pregnancies complicated by PE [48,62]. Therefore, we used target genes of 17 miRNAs (miR-21, miR-122, miR-155, miR-181a, miR-125b, miR-210, miR-193b, miR-516b, miR-518b, miR-221, miR-520a, miR-141, miR-136, miR-29a, miR-152, miR-423, and let-7c) for downstream analysis.

### 3.3. MicroRNA–mRNA Networks in IUGR

Using RNA hybrid, we found potential targets of all miRNAs linked to IUGR using genes Lists 1 and 2. Interestingly, miR-4743, which is downregulated in the IUGR placenta, can potentially target 2493 genes from List 1 and 236 genes from List 2. On the other hand, miR-141, which is upregulated in both placenta and maternal circulation in IUGR pregnancies, can potentially target only nine genes from List 1 and none of the genes from List 2. The other two miRNAs that are upregulated in both placenta and maternal circulation are let-7d and miR-424. Let-7d can potentially target 185 genes from List 1 and 17 genes from List 2, whereas miR-424 can target 48 genes from List 1 and 4 genes from List 2. The potential target genes of all miRNAs associated with IUGR are provided in Tables S3 and S4.

Insulin-like growth factor 2 (IGF2) has a well-established role in fetal development [137,138]. IGF2 mRNA-binding proteins (IGF2BP1–3), interact with 5´-UTR of IGF2 and stabilize it or protect it from miRNA-mediated silencing [137,138,139]. Therefore, IGF2BPs are also critical for fetal growth and have high expression in most fetal organs during embryogenesis [140]. Of all IUGR-linked miRNAs, IGF2 is targeted by four different miRNAs (miR-224, miR-370, miR-4743, and miR-574), IGF2BP1 is targeted by seven different miRNAs (miR-27a-star, miR-3679, miR-370, miR-4743, miR-5189, miR-623, and miR-664b), IGF2BP2 is targeted by two miRNAs (miR-373 and miR-4743), and IGF2BP3 is targeted by two miRNAs (miR-27a-star and miR-373). Similarly, several other target genes of IUGR-linked miRNAs are directly or indirectly associated with IGF-axis and/or fetal growth.

Due to evidence of upregulation of miR-141, miR-424, and let-7d in both placenta and maternal circulation during IUGR, these miRNAs are most suitable to be used as biomarkers of IUGR. Additionally, each of miR-210 and miR-193b was reported to be upregulated in the placenta during IUGR in three independent studies. Therefore, we used target genes of five IUGR-linked miRNAs (miR-141, miR-424, let-7d, miR-210, and miR-193b) for downstream analysis.

### 3.4. Pathways and Biological Processes Affected by Potential Biomarkers of PE

**Figure 3:**
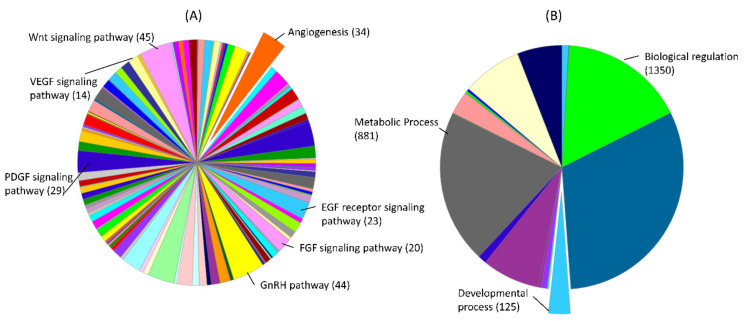
Pathways (**A**) and gene ontologies for biological processes (**B**), derived from Protein Analysis Through Evolutionary Relationships (PANTHER) classification system (v.16.0), and the number of genes associated with them. The target genes of 17 PE-linked miRNAs from genes List 1 were used in this analysis. A complete list of pathways and biological processes and genes linked with labeled pathways or biological processes is provided in Table S6.

We investigated the pathways and biological processes regulated by target genes of the 17 miRNAs that have more potential of serving as biomarkers. Collectively, these 17 miRNAs can target 2405 different genes from List 1 and we used the PANTHER classification system for evaluating the enrichment of these genes in different pathways and biological processes. Out of these target genes, 14 genes are involved in VEGF signaling and 34 genes are involved in angiogenesis (Figure 3A; Table S6). However, angiogenesis is not the only process dysregulated by these miRNAs, but it is the key process that can lead to severe placental pathologies including preeclampsia. Wnt signaling pathway has been well described for its role in trophoblast function and pathogenesis of PE [141]. Out of target genes of 17 PE-related miRNAs, 45 genes are involved in the Wnt signaling pathway (Figure 3A; Table S6). Fibroblast growth factor (FGF) is abundantly expressed in trophoblast cells and regulates progesterone synthesis and angiogenesis in the placenta [142]. Gonadotropin-releasing hormone (GnRH) also plays a vital role in placental angiogenesis by regulating the expression of pro-angiogenic chemokines in trophoblast cells [143]. Out of target genes of 17 PE-related miRNAs, 20 genes are involved in the FGF signaling pathway and 44 genes are involved in the GnRH pathway (Figure 3A; Table S6). Another interesting pathway regulated by these target genes is epidermal growth factor receptor (EGFR) signaling, which is required for fetal growth [144]. Out of target genes of 17 PE-related miRNAs, 23 genes are involved in EGF receptor signaling (Figure 3A; Table S6). Similarly, 29 genes are involved in platelet-derived growth factor (PDGF) signaling, which regulates several pregnancy-related processes including angiogenesis and post-implantation organogenesis [145]. Our analysis further shows that 881 genes are enriched in metabolic processes and 125 genes are enriched in developmental processes (Figure 3B). These findings suggest that the genes regulated by PE-related miRNAs regulate angiogenesis and can affect fetal growth and development. This might explain the frequent PE cases that are accompanied by impaired fetal development. All other pathways regulated by PE-related miRNA target genes are listed in Table S6.

**Figure 4:**
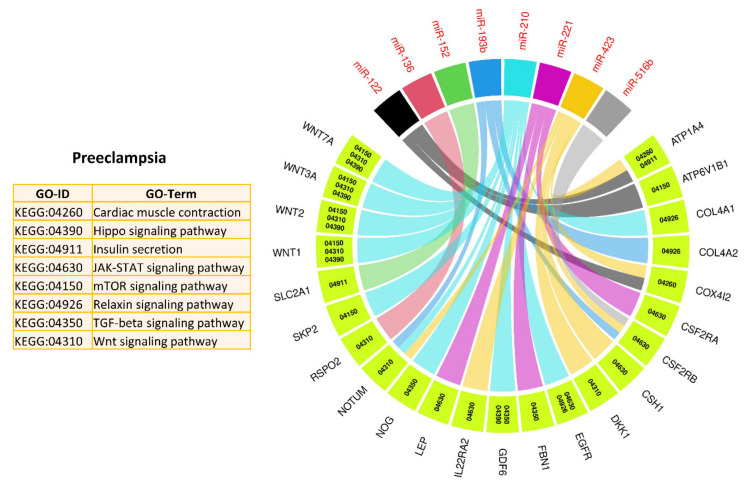
Kyoto Encyclopedia of Genes and Genomes (KEGG) pathways enrichment analysis of target genes of PE-linked miRNAs from genes in List 2, derived using ClueGO (version 2.5.1) and Cluepedia (version 1.5.7) plugin in Cytoscape (version 3.8.2) environment. A complete list of KEGG pathways regulated by PE-linked miRNAs is provided in Table S7.

**Figure 5:**
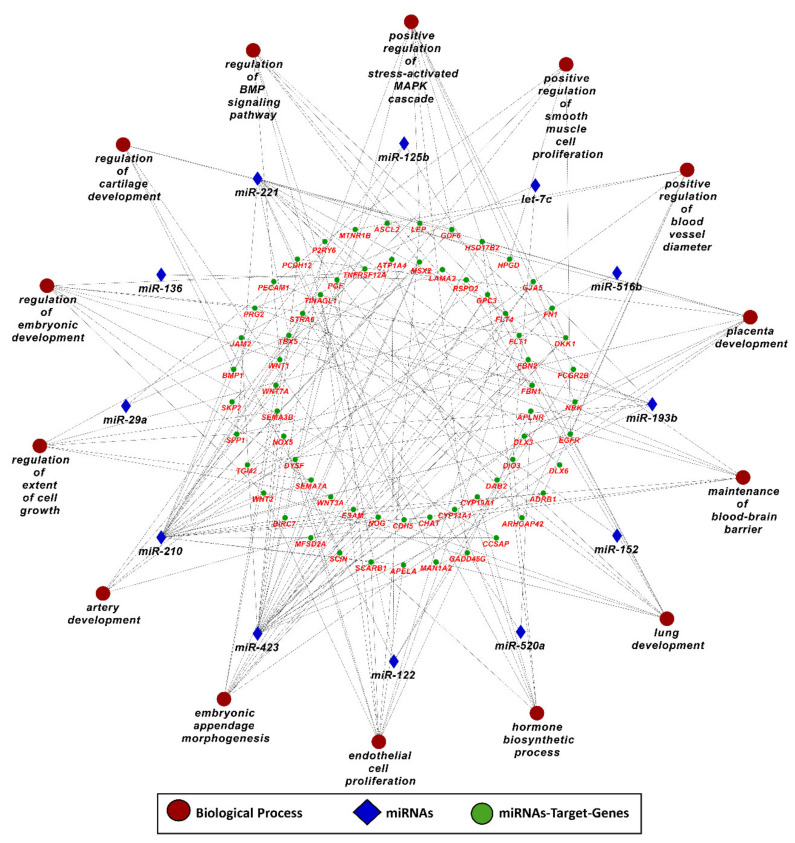
Gene ontology enrichment analysis for biological processes of target genes of PE-linked miRNAs from genes List 2, derived using ClueGO (version 2.5.1) and Cluepedia (version 1.5.7) plugin in Cytoscape (version 3.8.2) environment. A complete list of biological processes regulated by PE-linked miRNAs is provided in Table S7.

In the next step, we used target genes of 17 PE-related miRNAs from genes List 2 (elevated in the placenta) and performed downstream enrichment analysis for KEGG pathways and gene ontologies for biological processes using ClueGO (version 2.5.1) and Cluepedia (version 1.5.7) plugin in Cytoscape (version 3.8.2) environment (Figure 4 and Figure 5). PE-related miRNAs can potentially target 234 genes from List 2. By targeting different genes, PE-linked miRNAs regulate important KEGG pathways, including mammalian target of rapamycin (mTOR) signaling pathway, transforming growth factor beta (TGF-beta) signaling pathways, Janus kinase/signal transducers and activators of transcription (JAK–STAT) signaling pathway, Hippo signaling pathway, and Wnt signaling pathway (Figure 4). Mammalian target of rapamycin (mTOR) signaling pathway is a well-described pathway in the placenta and is involved in several important processes including invasion and migration of trophoblast cells, nutrients and oxygen transport, and hormone synthesis [146]. Similarly, transforming growth factor beta (TGF-beta) plays a vital role in placental development by stimulating differentiation of trophoblast cells to multinucleated lineage [147]. Janus kinase–signal transducers and activators of transcription (JAK–STAT) pathway is one of the main signaling mechanisms for a variety of growth factors and cytokines [148]. It regulates several important processes including cell proliferation, migration, mammary gland development, growth, and immune development [148]. Other than playing a vital role in trophoblast proliferation and differentiation, the Hippo pathway controls organ size, cell fate decision, stemness, and tissue regeneration [149,150]. Wnt/β-catenin signaling pathway is involved in the pathophysiology of severe PE through regulation of trophoblast proliferation and invasion [151]. Moreover, target genes of PE-related miRNAs are also enriched in several important biological processes including placenta development, embryo appendages morphogenesis, regulation of embryonic development, regulation of extent of cell growth, and artery development (Figure 5), which are critical for normal fetal development. A complete list of KEGG pathways and biological processes regulated by PE-linked miRNAs is provided in Table S7.

### 3.5. Pathways and Biological Processes Affected by Potential Biomarkers of IUGR

Five IUGR-linked miRNAs (miR-141, miR-424, let-7d, miR-210, and miR-193b) can target 1423 different genes from genes in List 1. First, we used these potential target genes for further downstream analysis using the PANTHER classification system. Downstream analysis of these genes shows that they are enriched in almost the same pathways and biological processes as in PE (Table S6). Other than regulating angiogenesis, EGFR signaling, and PDGF signaling, miRNA target genes in both PE and IUGR are also involved in developmental processes and metabolic processes (Figure 3, Table S6). Regulation of similar pathways in both PE and IUGR indicates that the root causes of placental pathologies in these disorders might be very similar, but have different physiological effects on mother and fetus, which is yet to be explained.

**Figure 6:**
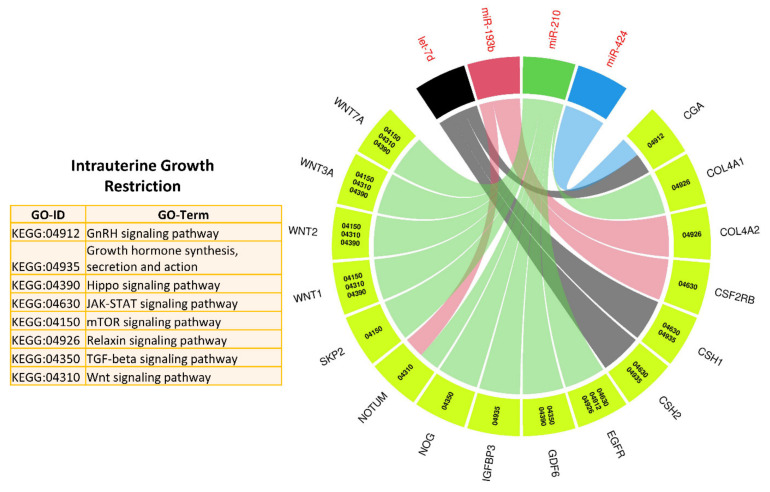
KEGG pathways enrichment analysis of target genes of IUGR-linked miRNAs from genes List 2, derived using ClueGO (version 2.5.1) and Cluepedia (version 1.5.7) plugin in Cytoscape (version 3.8.2) environment. A complete list of KEGG pathways regulated by IUGR-linked miRNAs is provided in Table S7.

**Figure 7:**
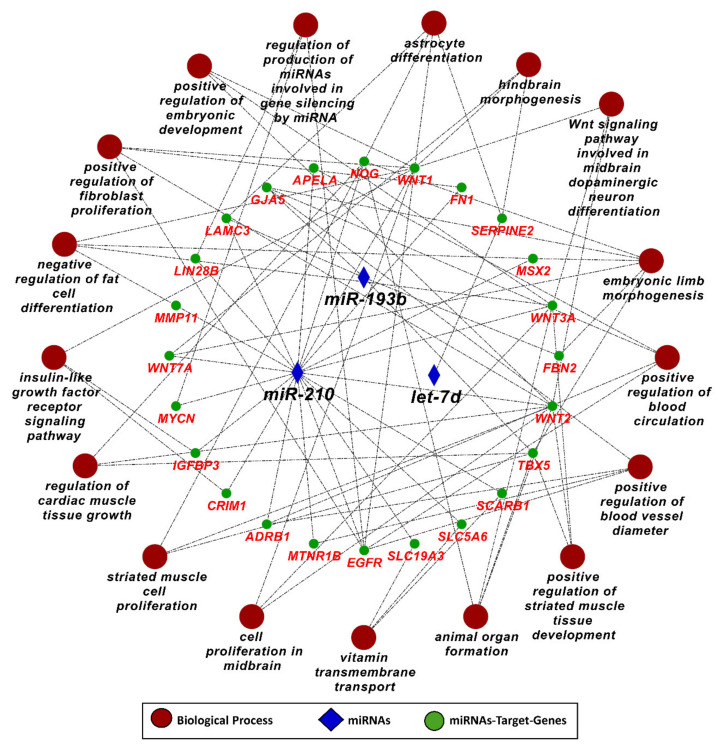
Gene ontology enrichment analysis for biological processes of target genes of IUGR-linked miRNAs from genes List 2, derived using ClueGO (version 2.5.1) and Cluepedia (version 1.5.7) plugin in Cytoscape (version 3.8.2) environment. A complete list of biological processes regulated by IUGR-linked miRNAs is provided in Table S7.

In the next step, we used target genes of five IUGR-related miRNAs from genes in List 2 (elevated in the placenta) and performed downstream enrichment analysis for KEGG pathways and gene ontologies for biological processes using ClueGO (version 2.5.1) and Cluepedia (version 1.5.7) plugin in Cytoscape (version 3.8.2) environment (Figure 6 and Figure 7). Four IUGR-related miRNAs (let-7d, miR-424, miR-210, and miR-193b) can potentially target 127 different genes from List 2, while miR-141 does not target any gene from this list. The target genes of the four IUGR-related miRNAs are enriched in important KEGG pathways including growth hormone synthesis and secretion, mTOR signaling pathway, GnRH signaling, Wnt signaling pathway, JAK/STAT signaling pathway, and Hippo signaling pathway (Figure 6). By regulating different genes, the IUGR-linked miRNAs also regulate several important biological processes including embryonic development, regulation of blood vessel diameter, astrocyte differentiation, organ morphogenesis, insulin-like growth factor receptor signaling pathway, and cardiac muscle growth (Figure 7). A complete list of KEGG pathways and biological processes regulated by IUGR-linked miRNAs is provided in Table S7. These findings suggest that, in pregnancies complicated by PE and IUGR, fetal growth can be affected either due to malfunctioning of the placenta or due to the direct effect of differentially expressed miRNAs on the growth and development of different fetal organs.

### 3.6. MicroRNA-210

MicroRNA-210 is one of the most studied miRNAs in recent years and has been mainly described as the principal miRNA induced under hypoxia, which regulates mitochondrial metabolism, cell proliferation, DNA damage response, and angiogenesis [152]. Hypoxia is considered as a signal that guides placental development by regulating several molecular signals vital for normal placentation [153]. It is an interesting analogy that most of the placental development occurs during the first trimester of pregnancy, which is a period of low oxygen tension in the placenta, and miR-210 is a hypoxia-responsive miRNA [154]. In this regard, the finding capturing attention is that 15 different studies showed dysregulation of miR-210 in PE, and 3 studies showed its dysregulation in IUGR (Table S5). Another important property of miR-210 is the wide range of its potential targets predicted in this study. MicroRNA-210 can target 968 genes from List 1 and 90 genes from List 2, meaning that miR-210 dysregulation can disrupt several important genetic pathways.

**Figure 8:**
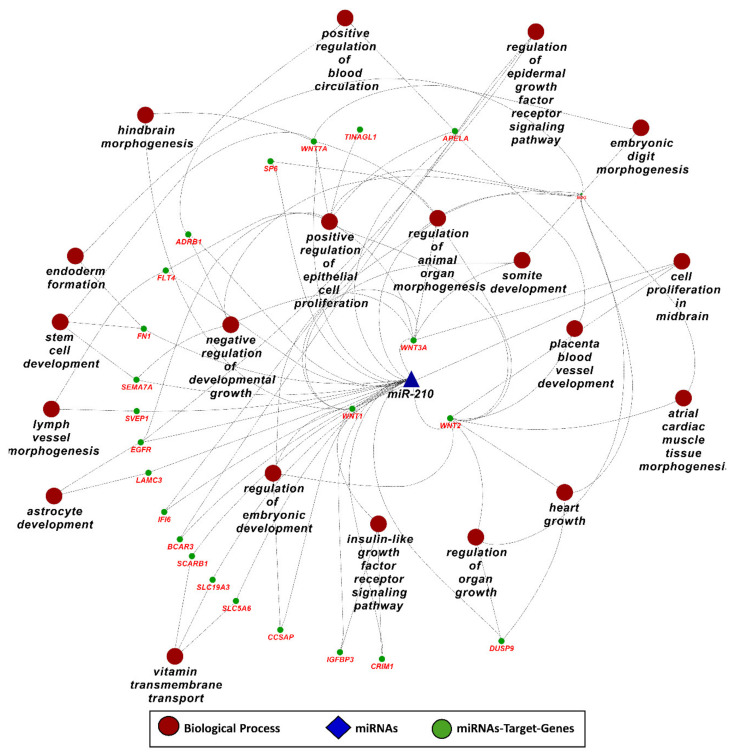
Gene ontology enrichment analysis for biological processes of miR-210 target genes from genes List 2, derived using ClueGO (version 2.5.1) and Cluepedia (version 1.5.7) plugin in Cytoscape (version 3.8.2) environment. A complete list of biological processes regulated by miR-210 is provided in Table S7.

Downstream analysis of miR-210 target genes from List 2, using ClueGO (version 2.5.1) and Cluepedia (version 1.5.7) plugin in Cytoscape (version 3.8.2) environment, shows that miR-210 can regulate important pathways and biological processes related to metabolism, growth, and development by targeting different genes (Figure 8). It can regulate organ growth by targeting Wnt family member 2 (WNT2), dual specificity phosphatase 9 (DUSP9), and noggin (NOG), embryonic digit morphogenesis by targeting NOG and WNT7A, placenta blood vessel development by targeting WNT2 and apelin receptor early endogenous ligand (APELA), astrocyte development by targeting laminin subunit gamma 3 (LAMC3), and epidermal growth factor receptor (EGFR) (Figure 8). A complete list of pathways and biological processes regulated by miR-210 is provided in Table S7. Previously determined functions, potential associations described in this study, and their frequent connection with placental pathologies make miR-210 a very promising biomarker for the diagnosis of reproductive disorders.

## 4. Conclusions

Pregnancy-associated disorders such as PE and IUGR can lead to fetal, newborn, and maternal morbidity and mortality. These complications are usually detected during the third trimester of pregnancy using conventional screening and diagnostic methods. In recent years, finding non-invasive early biomarkers of pregnancy complications has been one of the most studied topics in biomedical research. In this regard, microRNAs of placental origin have gained much attention, serving as potential diagnostic biomarkers for pregnancy complications. Placental miRNAs can be exported to both fetal and maternal compartments, but the physiological significance of these miRNAs in fetal and maternal compartments is unclear. If detectable in maternal circulation, an aberrant expression of placental miRNAs can be a prognostic tool to evaluate placental health and function and to predict the outcome of a pregnancy. Several studies have linked different miRNAs with the pathogenesis of PE and IUGR. This study investigated miRNAs that can be used as potential biomarkers of PE and IUGR, identified their target genes, and performed a comprehensive downstream analysis to clarify their role in the pathophysiology of PE and IUGR. We found that 17 miRNAs linked to PE and 5 miRNAs linked to IUGR have greater potential to be used as biomarkers. Target genes of these biomarker miRNAs are enriched in biological processes critical for normal placental and fetal development.

Although the role of several predicted target genes of PE- and IUGR-linked miRNAs have been previously described in the pathogenesis of these disorders, we also found some novel target genes from genes in List 2. All genes in this list are elevated in the placenta compared to all other tissues in the body [44]. Out of target genes of 17 PE-linked miRNAs from genes in List 2, 142 genes have not been previously associated with PE. Similarly, out of target genes of 4 IUGR-linked miRNAs, 81 genes have not been previously associated with IUGR. Out of these 81 target genes, HERV-H LTR-Associating 1 (HHLA1) is expressed only in the placenta and is targeted by miR-193b. All novel miRNA-mRNA pairs for PE and IUGR are listed in Table S8. This information can be used as a base to further investigate the role of these miRNAs in the pathogenesis of PE and IUGR and validate their target genes both in vitro and in vivo.

A large number of studies have linked dysregulation of certain miRNAs with the pathogenesis of pregnancy complications, but the existing data lack consistency. Different studies have linked different miRNAs with the same pregnancy complication, which also indicates the wide range of genetic pathways regulating placental development. These inconsistencies can be caused due to differences in gestational age at the time of sampling (first, second, or third trimester) and type of samples used (whole blood, serum, plasma, or placenta). Moreover, differential expression of a miRNA in the placenta might not reflect in maternal circulation and vice versa, which can result in a false-negative or false-positive diagnosis of placental health and pregnancy complications. Collecting samples and data at different time points throughout the gestation may be useful in finding reliable biomarkers for pregnancy complications. Nevertheless, scientific research has defined several miRNAs for their association with pregnancy disorders that can be used as a starting point for the future use of miRNAs in diagnostics and therapeutics.

## Figures and Tables

**Table 1:** Overview of miRNAs in preeclampsia (PE).

Name	Regulation	Source	References
let-7a, let-7c, miR-103, miR-122, miR-1233, miR-125a, miR-125b, miR-130b, miR-1323, miR-133a, miR-136, miR-141, miR-143, miR-152, miR-155, miR-181a, miR-182, miR-192, miR-193b, miR-21, miR-210, miR-215, miR-221, miR-24, miR-26a, miR-29a, miR-342, miR-423, miR-494, miR-495, miR-512, miR-515, miR-516a, miR-516b, miR-517b, miR-517c, miR-518b, miR-518e, miR-518f, miR-519a, miR-519d, miR-520a, miR-520c, miR-520d, miR-520g, miR-520h, miR-521, miR-525, miR-526b, miR-542, miR-574, miR-628, miR-629, miR-650, miR-758, miR-92a	Upregulated	Maternal circulation	[41,45,46,47,48,49,50,51,52,53,54,55,56,57,58,59,60,61,62,63,64,65,66,67]
miR-185, let-7d, miR-126, miR-1260, miR-1272, miR-144, miR-196b, miR-19b1, miR-223, miR-320c, miR-92a1, miR-766, miR-573, miR-409, miR-18a	Downregulated	Maternal circulation	[41,49,51,60,61,63,66,68,69]
let-7c, let-7d, miR-122, miR-125b, miR-134, miR-136, miR-141, miR-148a, miR-151, miR-152, miR-155, miR-16, miR-17, miR-181a, miR-193b-star, miR-20a, miR-20b, miR-21, miR-210, miR-221, miR-222, miR-25, miR-26b, miR-27a-star, miR-296, miR-29b, miR-30a-star, miR-31, miR-320a, miR-335, miR-362, miR-365a, miR-423, miR-431, miR-4421, miR-516b, miR-517-star, miR-518a, miR-518b, miR-519e, miR-520a, miR-524, miR-584, miR-638	Upregulated	Placenta	[10,45,62,66,70,71,72,73,74,75,76,77,78,79,80,81,82,83,84,85,86,87,88,89]
miR-542, miR-584, miR-17, miR-18a, miR-223, miR-19b1, miR-144, miR-126, miR-92a1, miR-1247, miR-204, miR-590, miR-1, miR-363, miR-150, miR-218, miR-32, miR-328, miR-625, miR-19a, miR-18b, miR-154, miR-411, miR-101, miR-195, miR-135b, miR-454, miR-374, miR-379, miR-149, miR-377, miR-10b, miR-450, miR-34c, miR-500, miR-139	Downregulated	Placenta	[66,73,87,88,90,91,92,93,94,95,96,97,98]

**Table 2:** Overview of miRNAs in intrauterine growth restriction (IUGR).

Name	Regulation	Where	References
let-7d, miR-103, miR-1306, miR-141, miR-148b, miR-16, miR-191, miR-200c, miR-205, miR-206, miR-224, miR-25, miR-27b, miR-27a-star, miR-30d, miR-335, miR-374a, miR-424, miR-432, miR-451, miR-491, miR-517a, miR-518b, miR-518e, miR-524, miR-93	Upregulated	Maternal circulation	[99,100,101,102,103,104]
miR-4454, miR-520a, miR-7975	Downregulated	Maternal circulation	[99,105]
let-7a, let-7b, let-7c, let-7d, let-7e, let-7f, let-7g, let-7i, miR-10b, miR-124, miR-141, miR-193b, miR-193b-star, miR-199a, miR-21, miR-210, miR-338, miR-34b, miR-363, miR-365a, miR-3679, miR-373, miR-424, miR-4287, miR-499a, miR-519a, miR-523, miR-572, miR-574, miR-590, miR-623, miR-664b, miR-758	Upregulated	Placenta	[10,106,107,108,109,110,111,112,113,114,115,116,117,118,119]
miR-526b, miR-5581, miR-519d, miR-519e, miR-520h, miR-515, miR-516b, miR-5189, miR-518b, miR-4535, miR-4743, miR-379-star, miR-380, miR-3622b, miR-370, miR-1323, miR-1, miR-105	Downregulated	Placenta	[113,118,120]

## Supplementary Materials

The following are available online at https://www.mdpi.com/1422-0067/22/5/2313/s1.
